# ICAM1 expression is induced by proinflammatory cytokines and associated with TLS formation in aggressive breast cancer subtypes

**DOI:** 10.1038/s41598-018-29604-2

**Published:** 2018-08-06

**Authors:** Stine L. Figenschau, Erik Knutsen, Ilona Urbarova, Christopher Fenton, Bryan Elston, Maria Perander, Elin S. Mortensen, Kristin A. Fenton

**Affiliations:** 1 RNA and Molecular Pathology Research group, Department of Medical Biology, Faculty of Health Science, UiT – The Arctic University of Norway, N-9037 Tromsø, Norway; 2 Tromsø University Proteomics Platform, Department of Medical Biology, Faculty of Health Science, UiT – The Arctic University of Norway, N-9037 Tromsø, Norway; 3 The Microarray Platform, Faculty of Health Science, UiT – The Arctic University of Norway, N-9037 Tromsø, Norway; 4 Histopath Diagnostic Specialists, Suite 201, 4 Drake Avenue, Building B Pinnacle Office Park, Macquarie Park, NSW 2113 Australia; 5 Department of Clinical Pathology, University Hospital of North Norway, N-9038 Tromsø, Norway

## Abstract

Intratumoral formation of tertiary lymphoid structures (TLS) within the tumor microenvironment is considered to be a consequence of antigen challenge during anti-tumor responses. Intracellular adhesion molecule 1 (ICAM1) has been implicated in a variety of immune and inflammatory responses, in addition to associate with triple negative breast cancer (TNBC). In this study, we detected TLS in the aggressive tumor phenotypes TNBC, HER2+ and luminal B, whereas the TLS negative group contained solely tumors of the luminal A subtype. We show that ICAM1 is exclusively expressed in TNBC and HER2 enriched subtypes known to be associated with inflammation and the formation of TLS. Furthermore, cell from normal mammary epithelium and breast cancer cell lines expressed ICAM1 upon stimulation with the proinflammatory cytokines TNFα, IL1β and IFNγ. ICAM1 overexpression was induced in MCF7, MDA-MB-468 and SK-BR-3 cells regardless of hormone receptor status. Taken together, our findings show that ICAM1 is expressed in aggressive subtypes of breast cancer and its expression is inducible by well-known proinflammatory cytokines. ICAM1 may be an attractive molecular target for TNBC, but further investigations elucidating the role of ICAM1 in targeted therapies have to take into consideration selective subtypes of breast cancer.

## Introduction

Breast cancer is the most common cancer among women and the leading cause of female cancer-related death^1^. Breast cancer is a heterogeneous disease, with large variation in cancer survival that shows substantial genetically and clinically disparity^2^. In breast cancer, as well as other cancers, the different types of immune cells interacting with each other and the tumor cells play key roles in shaping the microenvironment within and surrounding the tumor^3^. The concept of reciprocity between inflammation and cancer has been widely described^3–5^. Breast cancer often displays a high amount of different subpopulations of infiltrating immune cells. The tumor-associated lymphocytes appear to have a prognostic significance and are associated with better survival of breast cancer patients^6,7^. However, it remains unclear to what extent the composition of infiltrating immune cells differs and influences the clinical impact of the molecular subtypes of breast cancer. Molecular classification of breast cancer has become increasingly implemented as a supplement to the currently used morphological classification model^8–10^. Here, breast cancer is classified into the subtypes luminal A, luminal B, HER2 enriched, basal-like, normal breast-like and claudin-low. Triple negative breast cancer (TNBC) is defined by the absence of both hormone receptors, as well as HER2 expression, and constitutes a heterogeneous subtype that has a relatively poor clinical outcome. The majority of TNBC are of a basal-like subtype and is associated with high influx of lymphocytes^11,12^. Both basal-like and HER2 overexpressing breast cancer are thought to be more immunogenic as compared to luminal A carcinomas^13^.

Inflammation within the tumor microenvironment has been found beneficial for the outcome of breast cancer patients, in particular in cancers with functionally developed tertiary lymphoid structures (TLS)^14^. In TNBC, high levels of inflammation and adjacent TLS has been shown to be a prognostic factor for overall survival^15^. However, the majority of these patients do not achieve complete pathological response and no targeted treatment exists. Recently, it has been postulated that ICAM1 can serve as a therapy target for TNBC^16^. The intercellular adhesion molecule-1 (ICAM1/CD54), a transmembrane glycoprotein belonging to the immunoglobulin (Ig) superfamily, is expressed by several cell types including leucocytes, fibroblasts, and endothelial cells^17^. ICAM1 plays important roles in adhesion of cells, transendothelial migration of leucocytes to sites of inflammation, and activation of lymphocytes by interacting with lymphocyte function-associated antigen-1 (LFA-1, also known as ITGAL)^18^. Expression of ICAM1 in affected tissues is upregulated in response to a variety of inflammatory mediators and in autoimmune diseases^19,20^. Elevated levels of ICAM1 were reported in several malignancies^21–24^, where increased ICAM1 expression in breast, gastric, and colorectal cancers is correlated with more favorable prognosis^25–27^. Gene expression of ICAM1 differs between breast cancer subtypes, where it is found to be downregulated in luminal subtypes whilst upregulated in basal-like carcinomas^28^. The T cell rich areas of the TLS comprise high endothelial venules (HEVs) that express ICAM1, which represent a major gateway for lymphocyte migration into tumors^29^. The B cell follicle adjacent to the T cell zone, contains a network of CD21^+^ follicular dendritic cells (FDC) expressing ICAM1. The cell-cell adhesion of B cells to FDC is mediated by interaction between surface expression of ITGAL (LFA-1) and ICAM1, respectively^30^. It has been proposed that ICAM1 has an important function in antitumor immunity by being functionally involved in T cell priming by antigen-presenting cells, trans-endothelial trafficking of effector cells, and facilitating lymphocyte adhesion with tumor cells^31^. Conversely, ICAM1 has also been proposed to be involved in tumor cell invasion and migration into secondary sites^32–35^. Thus, the biological significance of ICAM1 expression in breast cancers remains controversial.

In this study, we have evaluated the ICAM1 expression in breast cancer. To clarify the expression profile of molecules important for lymphocyte trafficking, we investigated differentially expressed genes by SAGE-sequencing, where RNA was extracted from biopsies in paired samples taken from tumor, with and without TLS formation, and adjacent normal breast tissue. Additionally, we investigated the immunoreactivity of ICAM1 in normal breast tissue and tumor specimens, in addition to samples from a cohort of TNBC patients. Consequently, we evaluated ICAM1 expression in neoplastic cell lines, as well as in normal breast epithelial cell lines, and we surveyed proinflammatory cytokines for their potential to induce ICAM1 expression.

## Results

### ICAM1 is upregulated in breast carcinomas and associated with aggressive tumor phenotypes with TLS formation

**Figure 1:**
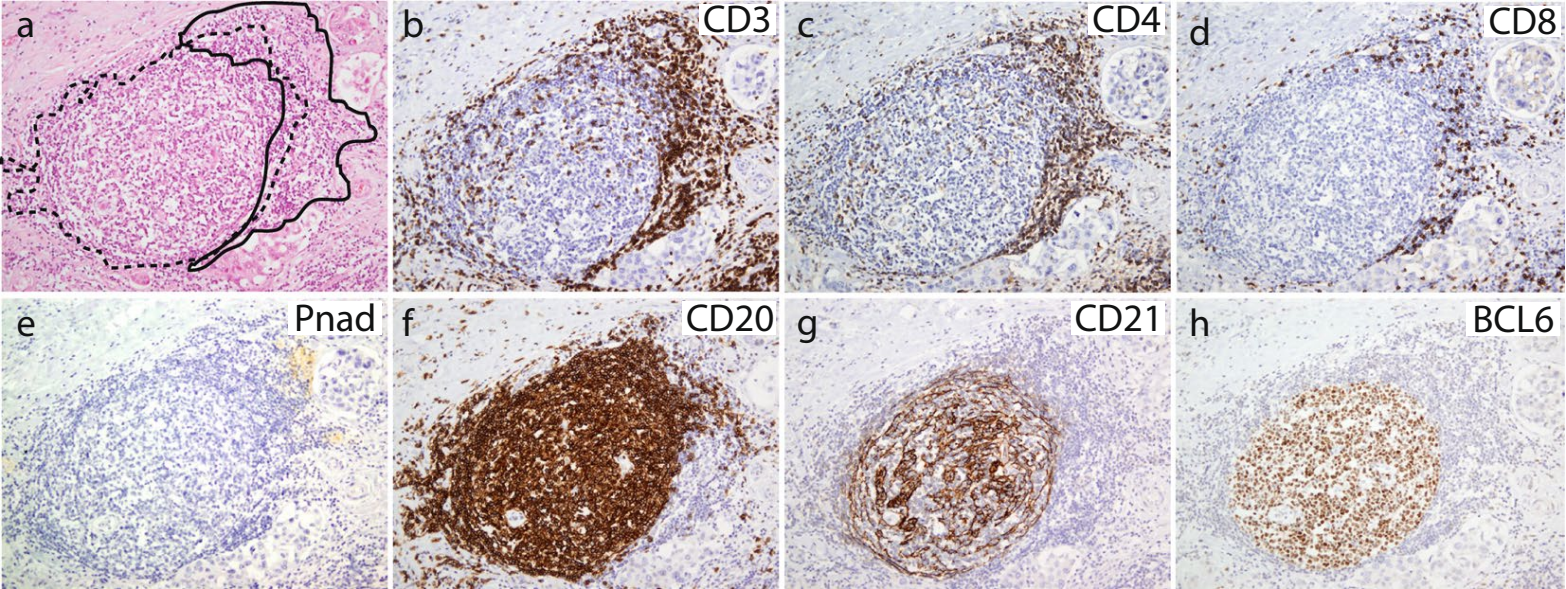
Intratumoral TLS formation in breast cancer patients share features of secondary lymphoid organs. Serial sections of tumor tissue showing the acquisition of features of secondary lymphoid organs with segregation of T cells and B cells into separate areas. (**a**) HE staining showing representative TLS formation with GC in breast tumor tissue. (**b**) CD3^+^, (**c**) CD4^+^, and (**d**) CD8^+^ T cells with development of (**e**) PNAd^+^ HEVs in the periphery of the T cell zone. (**f**) CD20^+^ B cells intermingled with a network of (**g**) CD21^+^ FDC that supports a functional germinal center response with (**h**) Bcl6 positive B cells. Solid black line: T cell area; dotted black line: B cell area. Original magnification, X100.

**Figure 2:**
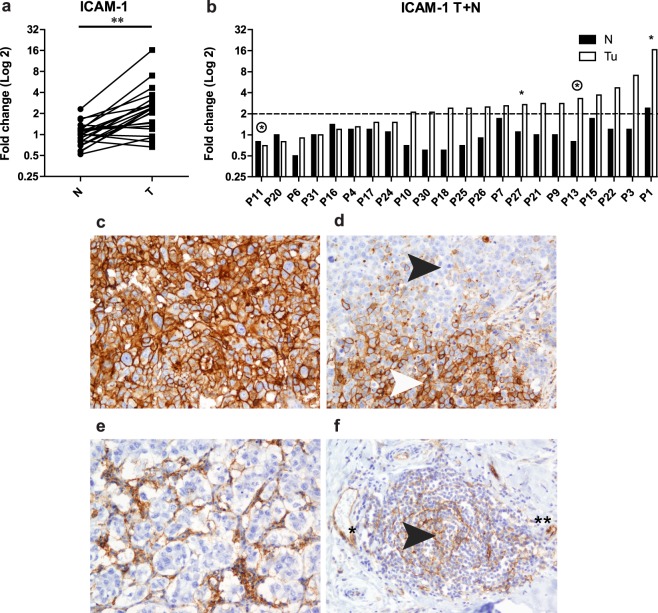
Expression of ICAM1 in breast cancer patients. (**a**) Gene expression level of ICAM1 in tumor and pair-matched normal breast tissue measured by quantitative PCR (qPCR). (**b**) Gene expression level of ICAM1 in paired tumor and normal samples. Tumor sections from triple negative breast cancer patients showed variable patterns of ICAM1 immunoreactivity. (**c**) Prominent ICAM1 staining of tumor cells, both in the periphery and central tumor areas. (**d**) Focally positive tumor cells (white arrow head; positive tumor cells, black arrowhead: negative tumor cells). (**e**) Negatively stained tumor cells with surrounding immune cells positively staining for ICAM1. (**f**) CD21^+^ FDC and HEVs expressing ICAM1 (black arrowhead and **, respectively). Original magnification, X200. N; normal tissue samples, T; tumor samples. Paired t test, **p < 0.01.

We previously demonstrated that intratumoral TLS formation is associated with higher levels of immune cell infiltration, hormone receptor status, and histological grade 3 tumors^36^. In the present study, we evaluated 23 surgically resected tumor biopsies from primary operable breast carcinomas with pair-matched normal tissue samples based on the organization of infiltrating lymphocytes, TLS formation, and ICAM1 expression. Patient clinicopathological parameters are presented in Table 1. Tumor samples were classified into TLS positive and TLS negative groups (Table 1) based on the detection of FDC in germinal centers (GC) within the B cell follicle and HEVs found in the T cell zone (Fig. 1). The T cell rich area consisted of clusters of CD3+ T cells (Fig. 1b–d) surrounded by PNAd+ HEVs (Fig. 1e). The B cell follicle comprised CD20^+^ B cells (Fig. 1f) and CD21^+^ FDC (Fig. 1g), with GC Bcl6^+^ B cells (Fig. 1h). The group of tumors with TLS formation were associated with a more aggressive tumor phenotype, and included subtypes such as TNBC, HER2 enriched, and luminal B, whereas the TLS negative group contained solely tumors of luminal A subtype (Table 1). We further assessed ICAM1 gene expression levels in the TLS positive and TLS negative groups of tumors by quantitative PCR (qPCR). The qPCR results from the 23 patient samples showed a significant upregulation of ICAM1 mRNA expression in tumors compared to normal tissue specimens (Fig. 2a, *p* < 0.01). Analyses of the ICAM1 immunoreactivity in these tumor specimens revealed positive staining in endothelial cells, lymphocytes in stroma, and macrophages. Interestingly, we detected ICAM1 positive tumor cells exclusively in triple negative and HER2 enriched subtypes (Table 1). The ICAM1 expression in these samples correlated to the individual mRNA expression in tumor and normal samples with the exception of one patient sample with HER2 enriched tumor (Fig. 2b). Interestingly, all tumors with ICAM1 positive tumor cells (n = 4) had high amount of immune cell infiltration and all were TLS positive (Fig. 2b and Table 1). In addition, we analyzed ICAM1 expression in tumor metastasis (n = 6) within dissected lymph nodes (LN) of these 23 patients. No positive ICAM1 staining of the tumor cells could be detected within the LNs (data not shown).

**Table 1:** Patients characteristics.

Patient number	ER	PR	HER2	Grade	LN status	Inflammation	TLS	ICAM1 Tu^a^	Subtype
SAGE_1T	na	na	na	dcis	neg	low	neg	neg	Dcis
SAGE_3T	neg	pos	pos	III	neg	high	pos	pos	HER2 enriched
SAGE_4T	pos	pos	neg	I	pos	low	neg	neg	Luminal A
SAGE_6T	pos	pos	neg	II	pos	low	neg	neg	Luminal A
SAGE_7T	pos	pos	neg	II	pos	high	pos	neg	Luminal B
SAGE_9T	pos	pos	neg	II	neg	high	pos	neg	Luminal A
SAGE_10T	pos	pos	neg	II	neg	low	neg	neg	Luminal A
SAGE_11T	neg	neg	pos	III	neg	high	pos	pos	HER2 enriched
SAGE_12T	pos	pos	neg	II	neg	high	pos	neg	Luminal A
SAGE_13T	neg	neg	neg	III	pos	high	pos	pos	TNBC
SAGE_15T	pos	pos	neg	II	neg	high	pos	neg	Luminal B
SAGE_16T	pos	pos	neg	II	pos	high	pos	neg	Luminal A
SAGE_17T	pos	pos	neg	I	neg	low	neg	neg	Luminal A
SAGE_18T	pos	pos	neg	I	neg	low	neg	neg	Luminal A
SAGE_20T	pos	pos	neg	II	neg	high	pos	neg	Luminal B
SAGE_21T	pos	pos	neg	I	neg	low	neg	neg	Luminal A
SAGE_22T	pos	pos	neg	III	neg	high	pos	neg	Luminal B
SAGE_24T	pos	pos	neg	I	neg	low	neg	neg	Luminal A
SAGE_25T	pos	pos	neg	I	neg	high	neg	neg	Luminal A
SAGE_26T	pos	pos	neg	I	neg	low	neg	neg	Luminal A
SAGE_27T	neg	neg	pos	II	neg	high	pos	pos	HER2 enriched
SAGE_30T	pos	pos	neg	I	pos	low	neg	neg	Luminal A
SAGE_31T	pos	pos	neg	I	neg	low	neg	neg	Luminal A

Abbreviations: DCIS: Ductal carcinoma *in situ*, ER: Estrogen receptor, HER2: Human epidermal growth factor receptor 2, Pos: Positive, PR: Progesterone receptor, na: not analysed, Neg: Negative, TLS: Tertiary lymphoid structures, TNBC: Triple negative breast cancer.

^a^>10% ICAM1 positive tumor cells.

### ICAM1 is overexpressed in triple negative breast cancer

As previously stated, ICAM1 overexpression has been associated with TNBC, and ICAM1 was suggested as a possible target for treatment in this patient group^16^. We therefore evaluated the ICAM1 protein expression by immunohistochemistry in a larger population of patients by including all 54 TNBCs diagnosed at UNN, Tromsø, Norway, from 2012 to 2015. Among the TNBC tumors tested, 63% of the samples showed positive ICAM1 staining in tumor cells (Table 2). Notably, the remaining triple negative tumors did not stain positively for ICAM1 in tumor cells, but all showed positive staining for ICAM1 expression in infiltrating immune cells. Among the positive cases of ICAM1 in tumor cells, the expression was predominantly detected in the invasive front of the tumor, whereas around one third showed prominent staining in the central tumor areas (Table 2). In all ICAM1 positive tumors, the protein was expressed in infiltrating immune cells in the periphery (Table 2).

**Table 2:** Characteristics of triple negative breast cancer patients.

ICAM1 tumor	n (%)
Negative	20 (37.0)
Positive	34 (63.0)
**ICAM1 central tumor**	
<10%	39 (72.2)
>10%	15 (27.8)
**ICAM1 periphery**	
<10%	20 (37.0)
>10%	34 (63.0)
**TLS status**	
Negative	14 (25.9)
Positive	40 (74.1)
**LN status**	
N0	41 (75.9)
N1	13 (24.1)
**Tumor grade**	
1	1 (1.8)
2	7 (13.0)
3	46 (85.2)
**Tumor size**	
<20 mm	31 (57.4)
>20 mm	23 (42.6)

Abbreviations: ICAM1: Intracellular adhesion molecule 1, LN: Lymph node, Neg: Negative, Pos: Positive, TLS: Tertiary lymphoid structures.

ICAM1 immunoreactivity was mainly localized in the cell membrane of the tumor cells (Fig. 2c and d), but different staining patterns of ICAM1 were observed. Some tumors showed pronounced staining throughout the tissue (Fig. 2c), whereas others had focally distributed staining for positive and negative ICAM1 (Fig. 2d). Tumor infiltrating lymphocytes stained always positively for ICAM1, both in the center of the tumor and the periphery (Fig. 2e). TLS within the tumors showed strong ICAM1 staining of FDC within GC, and HEVs located in areas surrounding the GC (Fig. 2f). ITGAL, the counterreceptor-ligand of ICAM1, was expressed in immune cells surrounding the tumor cells, both in the periphery and central tumor, as well as in the tumor-associated TLS (Supplementary Figure S1).

Importantly, neither tumors of positive hormone receptor subtype nor the normal epithelium of the breast showed any positive ICAM1 staining. No positive correlation could be found between lymph node status, tumor grade, and ICAM1 positive tumors (Table 2).

### Proinflammatory cytokines are potent inducers of ICAM1 expression in normal breast epithelial cells and breast cancer cell lines

**Figure 3:**
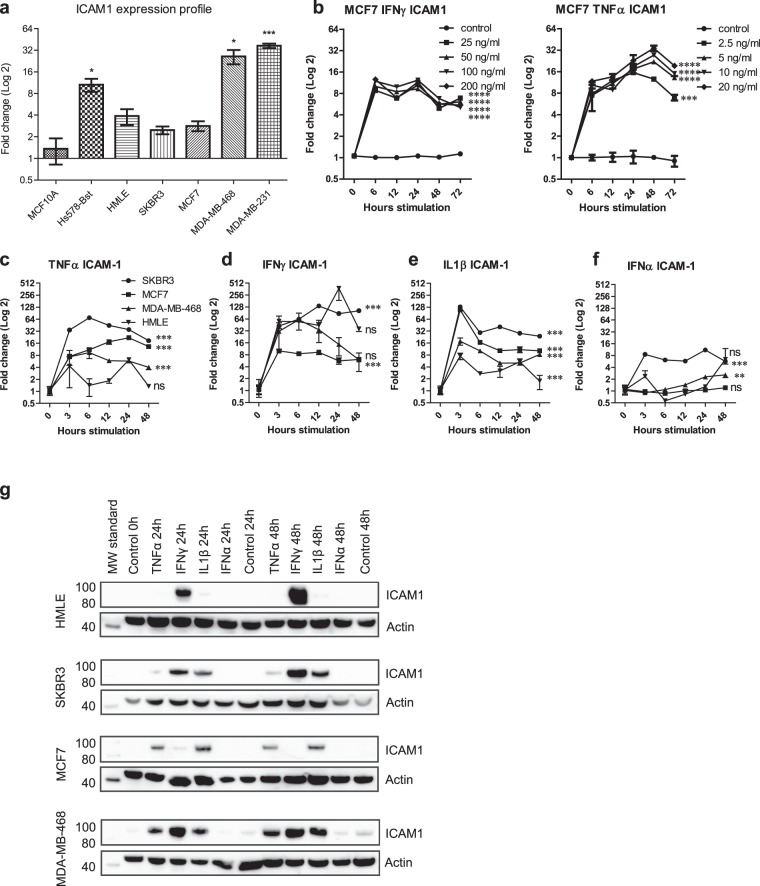
Expression of ICAM1 in human epithelial cells and breast cancer cells lines. (**a**) ICAM1 gene expression in breast cancer cell lines compared to a normal breast tissue cell line. (**b**) The expression of ICAM1 after stimulation of MCF7 cells with different concentrations of the proinflammatory cytokines IFNγ and TNFα were measured by qPCR. (**c**) Stimulation of TNFα induced the gene expression of ICAM1 in the breast cancer cell lines SK-BR-3, MCF7 and MDA-MB-468 and to a lesser extent in HMLE cells. (**d**) Stimulation by IFNγ. (**e**) Stimulation by IL1β. (**f**) Stimulation by IFNα. (**G**) Western blot analysis of ICAM1 protein levels in cell extracts from HMLE, SK-BR-3, MCF7 and MDA-MB-468 after stimulation with TNFα, IFNγ, Il1β and IFNα for 24 and 48 hours. Actin is shown as loading control. Full-length blots are available in Supplementary Figs 1–4 Two-way ANOVA, **p < 0.01, ***p < 0.001.

In the tumor microenvironment, proinflammatory cytokines can induce expression of other genes that are important for homing of immune cells and promoting inflammation. Since we observed a selective expression pattern of ICAM1 in our study, we wanted to investigate whether expression of ICAM1 in breast cancer cell lines could be induced by cytokines. First, we analyzed the basal expression profile of ICAM1 in different cell lines, both of cancerous origin: MDA-MB-231 *(ER*^*−*^*/PR*^*−*^*/HER2*^*−*^), MDA-MB-468 *(ER*^*−*^*/PR*^*−*^*/HER2*^*−*^), MCF7 *(ER*^+^*/PR*^+^*/HER2*^*−*^), SK-BR-3 *(ER*^*−*^*/PR*^*−*^*/HER2*^+^), and non-neoplastic cells: HMLE, Hs 578Bst, and MCF10A (Fig. 3a). Both TNBC cell lines, MDA-MB-231 and MDA-MB-468, showed the highest expression of ICAM1, with a 26- and 37-fold difference compared to MCF10A, respectively (*p* < 0.01) (Fig. 3a). In contrast, the expression of ICAM1 in MCF7 and SK-BR-3 cell lines was similar compared to MCF10A (Fig. 3a). Of the additional non-tumorigenic cell lines, Hs 578Bst and HMLE, only Hs 578Bst showed a significantly increased expression of ICAM1 compared to MCF10A (fc 10.6, *p* < 0.01). MCF7 cells were chosen for further dose dependent stimulation experiments based on the low basal expression level of ICAM1 and hormone receptor status. Both IFNγ and TNFα cytokines significantly induced ICAM1 mRNA expression, with peak levels reached after 24 hours (Fig. 3b). Subsequently, we examined all the neoplastic cell lines for ICAM1 expression after exposure to several cytokines (Fig. 3c–f). TNFα, IFNγ, and IL1β significantly increased the production of ICAM1 in both SK-BR-3, MCF7, and MDA-MB-468 cell lines after 24 hours in comparison to unstimulated basal conditions (Fig. 3c–e). Notably for IL1β, there was a pulse induction of ICAM1 expression after 3 hours followed by a second peak at 24 hours (Fig. 3e). In contrast, no significant induction of ICAM1 expression was observed upon IFNα stimulation for MCF7 and MDA-MB-468 cells over the 48-hour period examined, whereas an 8-fold induction was observed for SK-BR-3 cells (Fig. 3f). ICAM1 mRNA expression was induced with IFNγ in HMLE cells (Fig. 3d). TNFα and IL1β did also induce the ICAM1 mRNA expression, but to a lesser extent (Fig. 3c and e). At the protein level, the production of ICAM1 was detected in HMLE cells exposed to IFNγ after 24 and 48 hours, and a weak band was observed after IL1β stimulation at both time points (Fig. 3g). ICAM1 protein expression was also evident at 24 and 48 hours after IFNγ stimulation in SKBR3 and MDA-MB-468 cells, whereas in MCF7 cells only a weak band could be detected at 24 hour (Fig. 3g). Protein expression of ICAM1 after IL1β stimulations was detected in SK-BR-3, MCF7, and MDA-MB-468 cells (Fig. 3g). TNFα induced expression of ICAM1 in MCF7 and MDA-MB-468 cells after 24 and 48 hours, whereas SK-BR-3 cells showed only weak ICAM1 expression (Fig. 3g). Unstimulated MDA-MB-468 cells showed weak expression of ICAM1 at 0 hour that increased during the 24 and 48 hours incubation time (Fig. 3g). No increase in ICAM1 protein expression could be observed after IFNα induction in any of the cell lines. Altogether, these results show that each stimulant is a potent inducer of ICAM1, regardless of hormone receptor status and metastatic potential of the cells.

### Normal and malignant breast gene expression profiles

To investigate global gene expression patterns in tumor versus adjacent normal tissue, we performed a comparative analysis of differentially expressed genes from clinical biopsies of breast tumor and pair-matched normal tissue specimens (Table 1 and Supplementary Tables S1 and S2). SAGE data generated in total close to 1.3 billion reads (1,290,594,733 raw SAGE-seq reads), out of which approximately 67% were mapped to the human genome (Supplementary Table S3). In order to identify the gene expression differences between normal and tumor specimens, we employed a statistically stringent analysis. This analysis revealed 1,323 differentially expressed genes between normal and tumor specimens. Among these genes, 613 genes were upregulated, and 710 genes were downregulated in tumors (see Supplementary Table S4 for complete gene list).

### Comparison of differentially expressed genes in TLS positive and TLS negative breast tumors

**Figure 4:**
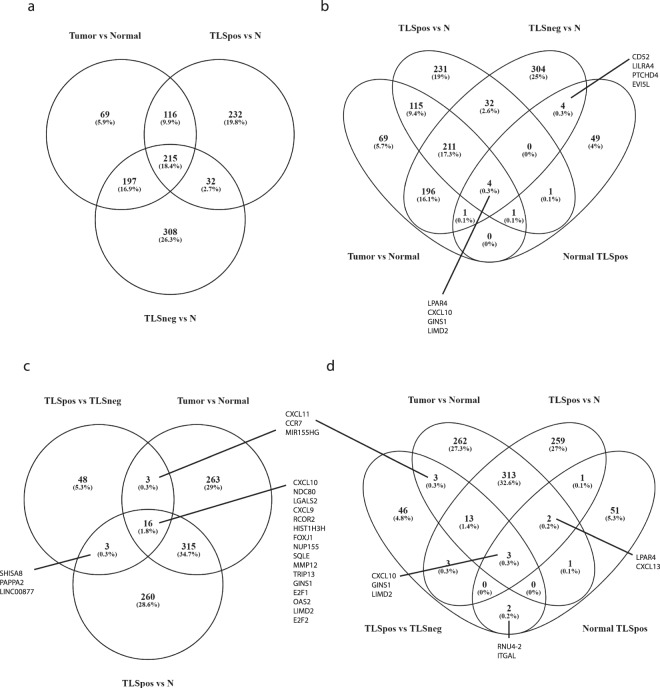
Differentially expressed genes in tumors with and without TLS formation compared to normal samples. Venn diagrams were generated to illustrate the number of upregulated genes differentially expressed in (**a**) Tumor versus Normal, TLS positive tumors versus Normal, and TLS negative tumors versus Normal. (**b**) TLS positive tumors versus Normal, TLS negative versus Normal, Tumor versus Normal, and Normal TLS positive (**c**) TLS positive tumors versus TLS negative tumors, Tumors versus Normal, and TLS positive versus Normal. N; Normal, TLSpos; TLS positive tumors, TLSneg; TLS negative tumors.

**Figure 5:**
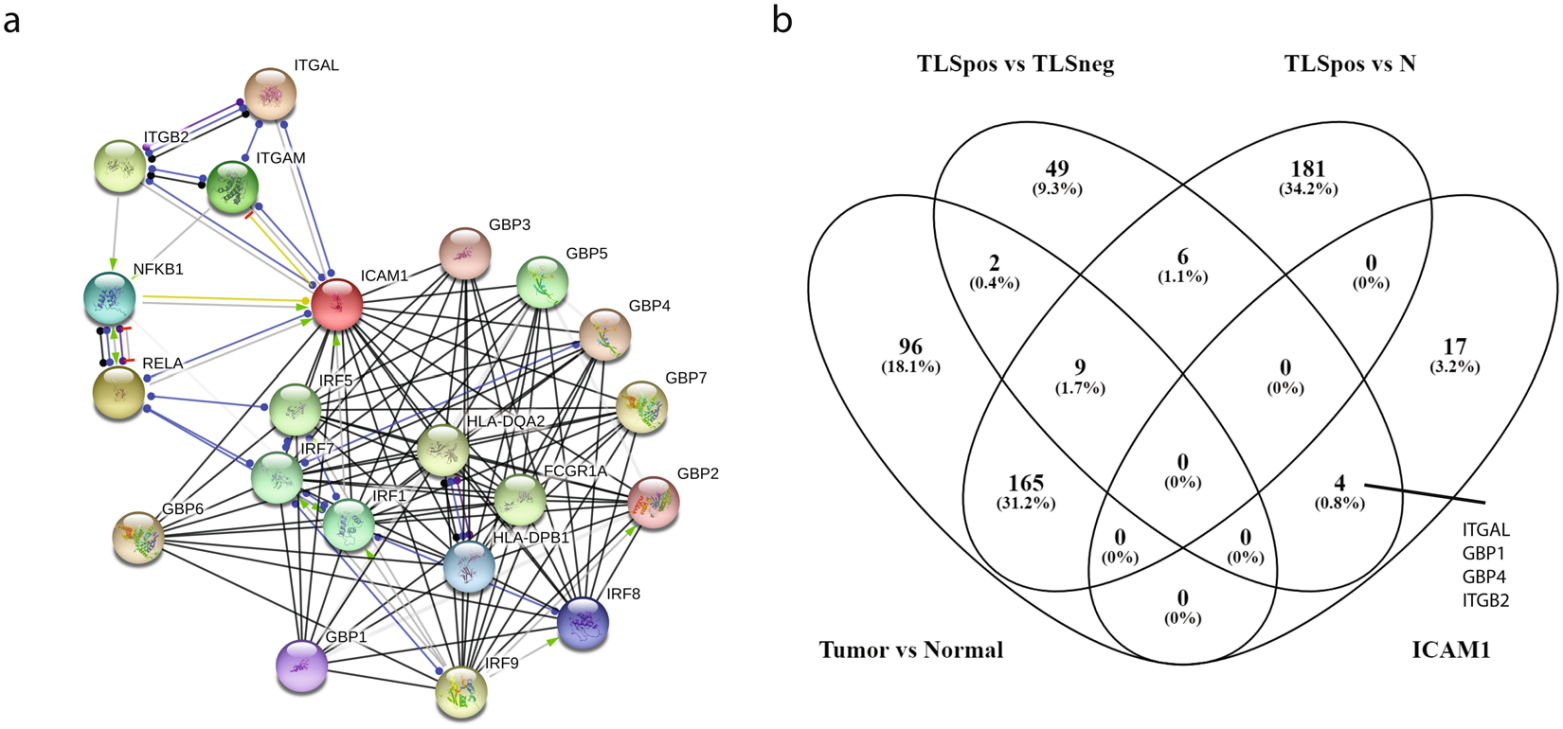
Identification of genes related to ICAM1 in TLS positive tumors. (**a**) An interaction node network of the nearest partners to ICAM1 obtained using STRING software. (**b**) Comparison of the differentially expressed genes from Tumor versus Normal, TLS positive tumors versus TLS negative tumors, TLS positive tumors versus Normal, and the interaction partners of ICAM1. N; normal, TLSpos; TLS positive tumors, TLSneg; TLS negative tumors.

In order to identify the most commonly occurring gene expression changes in the two tumor groups (TLS positive or TLS negative, Table 1), the tumors were profiled independently from the patient-matched normal tissue samples. We found 1,423 differentially expressed genes when comparing TLS positive tumor samples with paired normal tissue (N) samples. In the TLS positive group, we noted 604 upregulated and 819 downregulated genes (*p* < 0.05; see Supplementary Table S5 for complete gene list). In the TLS negative group, 1,807 transcripts were found to be differentially expressed, 790 genes were upregulated and 1,017 genes were downregulated between TLS negative samples and paired normal samples (N) (*p* < 0.05; see Supplementary Table S6 for complete gene list). The relationships between significantly upregulated genes among the pairwise comparisons of Tumor versus Normal samples, TLS positive versus paired normal samples (TLS positive versus Normal) and TLS negative versus paired normal samples (TLS negative versus Normal) are presented in a Venn diagram in Fig. 4a. The comparison revealed 215 upregulated genes in common that were found to be functionally enriched in different biological processes, e.g. mitotic cell cycle (GO:0000278), extracellular matrix disassembly (GO:0022617) and negative regulation of macromolecule metabolic process (GO:0010605) among others (Supplementary Table S9). Surprisingly, in the TLS positive versus Normal comparison, only a few genes were involved in immune responses. We therefore compared the differentially expressed genes of the normal samples taken adjacent to tumors with and without TLS. The comparative analysis revealed 155 genes, of which 60 were upregulated and 95 were downregulated in normal samples taken from paired TLS positive tumors compared to normal samples taken from paired TLS negative tumors (Supplementary Table S7 for complete gene list). Of the 60 genes with higher expression in normal samples from TLS positive tumors (Normal TLS positive), only 3 genes (LPAR4, CXCL10 and GINS1) were upregulated in all comparisons. One gene (CXCL13) was found upregulated in Tumor versus Normal, TLS positive versus Normal and Normal TLS positive, another gene (ICA1) was upregulated in Tumor versus Normal, TLS negative versus Normal and Normal TLS positive, and one more gene (CD52) was upregulated in TLS negative versus Normal and Normal TLS positive (Fig. 4b and Supplementary Table S10). Since the differentially expressed genes in normal samples taken from TLS positive and TLS negative tumors are known to be involved in immune responses, we performed a comparative analysis of genes expressed only in TLS positive and TLS negative tumors (Table 1 and Supplementary Table S2). Our comparative analysis revealed 163 genes that were differently expressed in tumors with and without TLS formation (see Supplementary Table S8 for complete gene list). Of these, 70 genes were upregulated and 93 were downregulated among the TLS positive tumors. Selected genes related to leukocyte migration that were upregulated in TLS positive tumors included CTLA4, ITGAL, CXCL9, CXCL10, CXCL11, CCR7, XCL1, and XCL2. Downregulated genes of interest in tumors with TLS formation were PGR, PIK3R1, and HMGB1 (Supplementary Table S8). A Venn diagram comparing the upregulated genes in TLS positive tumors (TLS positive versus TLS negative) with upregulated genes in the Tumors versus Normal comparison, and the TLS positive versus Normal comparison is shown in Fig. 4c. These comparisons revealed that only 16 genes were upregulated in all comparisons, three genes were common in TLS positive versus TLS negative and TLS positive versus Normal, three genes were common between TLS positive versus TLS negative and Tumor versus Normal, and 315 genes were upregulated in Tumor versus Normal and TLS positive versus N comparisons (Fig. 4c and Supplementary Table S11). We could not detect ICAM1 among the differentially expressed genes found in the different comparisons. We therefore used STRING to select the 20 nearest interaction partners (Fig. 5a). When comparing the genes from the Tumor versus Normal, TLS positive versus TLS negative and TLS positive versus Normal with the interaction partners of ICAM1, four genes were identified (Fig. 5b and Supplementary Table S12). ITGAL, GBP1, GBP2 and ITGB2 were all upregulated in TLS positive tumors compared to TLS negative, but no changes could be observed in Tumor versus Normal and TLS positive versus Normal (Fig. 5b). We also identified an upregulation of ITGAL in normal samples from TLS positive tumors compared to normal samples from TLS negative tumors (Supplementary Table S7).

## Discussion

Non-resolving inflammation is one of the defining features of the tumor microenvironment, in addition to involvement of proinflammatory mediators in cancer progression, such as adhesion molecules, chemokines, and cytokines. TLS are thought to act as a powerhouse of anti-tumor immunity by bringing the whole immune response machinery at the tumor site. We have previously been studying the intriguing observation that TLS forms within the tumor microenvironment in human breast cancer patients^36^. Our initial work focused on tumor infiltrating lymphocytes, how they were organized, and the association with clinicopathological features. We observed that breast carcinomas frequently contained TLS and that the presence of these structures was associated with a more aggressive tumor phenotype^36^. However, it is still unclear whether TLS are either a consequence of an immune response *per se* or sites of an active immune reaction against the components within the tumor microenvironment. Here we present findings that TLS positive tumors included the more aggressive subtypes, such as TNBC, HER2 enriched and luminal B, while tumors in TLS negative groups were all of luminal A subtype. In addition, we identified genes involved in important migratory and signaling events related to tumor-associated TLS formation to be expressed in TLS positive tumors.

ICAM1 is an important molecule for lymphoid trafficking and has been shown to be upregulated in several types of cancers, including breast cancer^24^. Our verification studies showed that ICAM1 mRNA was expressed at higher level in tumor compared to the adjacent normal breast tissue. Furthermore, immunohistochemical analyses revealed ICAM1 immunoreaction in tumor cells of more aggressive subtypes, including HER-2 enriched and triple negative tumors. Supporting this, Guo and colleagues detected ICAM1 expression solely in triple negative tumors and therefore proposed ICAM1 as a molecular target for TNBC^16^. TNBC is characterized by high influx of tumor-associated lymphocytes, an aggressive phenotype, and high metastatic potential of tumor cells. Tumor-localized TLS are associated with a more aggressive tumor phenotype and are frequently found in TNBC. Challenging this, in the cohort of triple negative patients approximately 60% of the samples had ICAM1 positive tumor cells. It is important to mention that the percentage of tumor cells that expressed ICAM1 ranged from less than 10 to 100% with intertumoral differences in staining patterns. Questioning the proposed TNBC-specific expression profile of ICAM1, we investigated whether a proinflammatory stimuli could mimic the milieu found in the tumor microenvironment and result in overexpression of ICAM1 also in hormone sensitive subtypes. We showed that ICAM1 expression could be induced by proinflammatory cytokines, both in hormone receptor positive cells and normal breast epithelial cells, in addition to the basal-like and HER2 positive cell lines.

A series of lines of evidences suggests that ICAM1 can potentially be involved in invasion of tumor cells and metastasis of human breast cancer^24^. Supporting our findings, Rosette and colleagues observed that the level of ICAM1 expression was positively correlated with the metastatic potential of cell lines, and they reported a trend towards elevated expression in tumor specimens compared to normal control tissues^37^. Furthermore, ICAM1 might be involved in cancer metastasis, as shown by silencing of ICAM1 in MCF7 breast cancer cells. This can attenuate the metastatic ability *in vitro* and result in decreased migration and invasion accompanied by a lower MMP14 expression^38^. It has also been demonstrated by exploiting ICAM1 overexpression in breast cancer cells, that ICAM1 may serve as an effective nanomedicine target by delivering siRNA to TNBC cells and inhibiting cancer progression^39^.

The migration of lymphocytes from blood vessels and TLS associated HEVs in into tissue is well characterized at a molecular level, in contrast to the emigration of tumor cells^40^. However, ICAM1 is hypothesized to facilitate the attachment of carcinoma cells to the lymphatic endothelium and therefore promote the micro-metastatic movement in regional lymph nodes. Furthermore, it has been shown that tumor cells utilize leukocytes as linker-cells to endothelium for their extravasation. Strell and colleagues demonstrated that tumor cells expressing ICAM1 interact with neutrophil granulocytes that facilitates the contact to the endothelium by β2-integrins^41^. The same interaction has been shown for IL-8 producing melanoma cells^42,43^. We demonstrated that ICAM1 expression was inducible in tumor cells upon pro-inflammatory stimuli which might be important for the extravasation process from the blood vessels. We speculate that ICAM1 expression can be upregulated by cytokines in the tumor microenvironment prior to intravasation into the circulation. The blood vessels that arise in the tumor are often immature and vulnerable that can result in leakage through the vessel wall and this allows the tumor cells to leave the tissue more easily. On the other hand, expression of ICAM1 by the tumor cells might lead to specific T cell recognition and enhancement of effector CTL adhesion that might not be in favor for the tumor cells. ICAM1/LFA-1 interactions have been proposed to play critical roles in enhancing antitumor immune responses^31,44^. Moreover, induced overexpression of ICAM1 in tumor cells have been shown to result in reduced tumor growth correlated with increased lysis by tumor infiltrating lymphocytes^45,46^. It is intriguing to speculate that it might be the cytokine production within the tumor microenvironment that causes the expression of ICAM1 in the tumor cells, since TNFα and IFNγ are known to induce synthesis of ICAM1^47^. However, the absence of ICAM1 expression in hormone receptor positive tumors remains to be elucidated and warrant more attention to this interesting phenomenon. Taken together, our findings show that ICAM1 is upregulated in more aggressive subtypes of breast cancer and its expression is inducible by well-known proinflammatory cytokines, regardless of hormone receptor status of the tumor cells. ICAM1 may still be an attractive molecular target for TNBCs, but further investigations elucidating the role of ICAM1 in targeted therapies have to take into consideration selective subtypes of breast cancer.

## Methods

### Patients

The study was approved by the Regional Committees for Medical and Health Research Ethics (REC; Norway, 2010/1523). Tumor and normal tissue specimen for SAGE-sequencing were collected from 23 patients that were operated at the University hospital of North Norway (UNN) in Tromsø in 2012. A written informed consent was obtained from all subjects and all methods were carried out in accordance with relevant guidelines and regulations. The main clinical and pathological parameters of the included patients are presented in Table 1. Full-faced hematoxylin and eosin (HE) stained sections were used to assess the degree of infiltrating immune cells in the breast tumors as described previously^36^. Histological tumor grade was assessed by the Nottingham Grading System^48^. The cut off values for Estrogen (ER) and Progesterone (PR) were 10%. Tumors demonstrating HER2 protein overexpression or amplified *HER2* gene (IHC 3+ or FISH *HER2* gene ratio ≥2) were considered to be positive. None of the included patients received adjuvant therapy before surgery, nor did they have any other known malignancies.

### Immunohistochemistry

54 triple negative breast carcinomas (TNBC), 30 tumors with different hormone receptor status, and 30 normal breast tissue samples were scored for ICAM1 staining. We used archived (2012–2015) formalin-fixed paraffin embedded (FFPE) specimen obtained from the Department of Clinical Pathology (UNN, Tromsø). ICAM1 expression was defined as follows: negative, <10% of tumor cells showing membranous staining; positive >10% of tumor cells showing membranous staining, both in the center of tumor and in the periphery. In addition, tumor and normal breast tissue sections from biopsies (n = 23) used for SAGE-sequencing and dissected lymph nodes (n = 6) from the same patients were evaluated for ICAM1 expression. Immunohistochemistry was performed on FFPE sections using Envision+ System-HRP(DAB+) detection kit (K4011, Dako), Polink-2 Plus HRP Goat with DAB kit (D43-18, GBI Labs), or platform-specific assays on BenchMark XT (Ventana Medical systems Inc., USA) as described previously^36^. Anti-Bcl6 [GI191E/A8] (760-4241, Ventana), anti-CD3 [2GV6] (790-4341, Ventana), anti-CD4 [SP35] (790-4423, Ventana), anti-CD8 [SP57] (790-4460, Ventana), anti-CD20 [L26] (760-2531, Ventana), anti-CD21 [EP3093] (ab75985, Abcam), anti-ICAM1 [EP1442Y] (ab53013, Abcam), anti-ITGAL/CD11a (LS-B5915, LifeSpan BioSciences, Inc), and anti-PNAd [MECA-79] (120801, Biolegend) antibodies were used detection of intratumoral TLS. Normal human tonsil was used as a positive control for ICAM1 immunoreactivity. Images were required using Olympus BX51 microscope operated with Cell-F software (Olympus).

### Cell cultures and cytokine stimulation

The following human breast cancer cell lines were obtained from the ATCC: Hs 578Bst, MCF7, MCF10A, MDA-MB-231, MDA-MB-468, and SK-BR-3. HMLE cells were a gift from Robert Weinberg, Whitehead Institute for Biomedical Research and Department of Biology, Massachusetts Institute of Technology. Cells were cultured in MEM (M4655, Sigma-Aldrich) or RPMI-1640 (R8758, Sigma-Aldrich) supplemented with 10% fetal bovine serum (FBS) (S0615, Biochrom GmbH), 1% penicillin-streptomycin (P0781, Sigma-Aldrich), and to MCF7 cells was added 0.01 mg/ml insulin (I9278, Sigma-Aldrich). MCF10A cells were cultured in DMEM/F12 (ThermoFisher Scientific), supplemented with 5% horse serum (ThermoFisher Scientific), 20 ng/ml EGF, 0.5 μg/ml hydrocortisone (Sigma-Aldrich), 100 ng/ml cholera toxin (Sigma-Aldrich), and 10 μg/ml insulin. HMLE cells were grown in a 1:1 mixture of MEBM (Lonza) with DMEM/F12 (Sigma-Aldrich) supplemented with 10 ng/ml EGF 0.5 μg/ml hydrocortisone 0.01 mg/ml insulin, and 1% penicillin-streptomycin. All cell lines were incubated in a humidified atmosphere of 5% CO_2_ at 37 °C. Media was changed every third day and cells were cultured up to 80% confluence before washing with phosphate-buffered saline (PBS) (D8537, Sigma-Aldrich) pH 7.4, followed by 0.25% Trypsin-EDTA solution (T4049, Sigma-Aldrich) treatment. Before stimulation, cell lines were cultured overnight in six-well-plates (2.0 × 10^5^) under starved conditions. Cells were stimulated with the following recombinant human cytokines: IFNγ 50 ng/ml (285-IF-100, R&D systems), TNFα 5 ng/ml (210-TA-100, R&D systems), IL1β 20 ng/ml (201-LB-025, R&D systems) or IFNαA/D 16 ng/μl (I4401-100KU, Sigma-Aldrich) for 3, 6, 12, 24 and 48 hours in culturing media. Controls were un-stimulated cells cultivated with normal seeding media.

### RNA isolation, cDNA synthesis, and quantitative real-time polymerase chain reaction

Cell cultures were washed twice with PBS pH 7.4 and harvested with 1 ml TRIsure™ reagent (BIO-38033, Bioline) followed by total RNA extraction. Quantity and purity of the extracted RNA was determined using the NanoDrop 2000 (ThermoFisher Scientific). Isolated RNA from cell cultures and the remaining RNA from SAGE library preparations were synthesized into cDNA using High-capacity cDNA Reverse Transcription Kit (4368814, ThermoFisher Scientific) according to manufacturer instructions. Real-time PCR was performed using the following TaqMan (ThermoFisher Scientific) probe assays: ACTB (Hs01060665_g1), GAPDH (Hs03929097_g1), ICAM1 (Hs00164932_m1), PRLP0 (Hs99999902_m1). Total cDNA input was normalized to reference genes measured in parallel PCR reactions. Real-time qPCR data is presented as relative expression using ΔΔCT method in which relative expression = 2^−ΔΔCT ^^49^. Each sample was run in duplicates and threshold cycle (C_q_) values were averaged from each reaction. ACTB, GAPDH, and RPLP0 were used as reference genes.

### Western blotting

All cell lines were collected with 1X NuPage^®^LDS Sample Buffer (NP0007, ThermoFisher Scientific). All cell lysates were boiled and sonicated before proteins were separated by SDS-PAGE 4–12% Bis-Tris gels (NP0323, ThermoFisher Scientific) according to manufacturers’ protocol. Proteins were blotted onto PVDF membranes (IPVH00010, Merck Life Science). Membranes were blocked in 5% dry milk powder/TBS-T (170-6404, Bio-Rad), washed and incubated overnight at 4 °C with monoclonal anti-ICAM1 antibody (ab53013, Abcam) diluted 1:2000. Detection was performed with goat anti-rabbit IgG (H + L) antibody (65-6120, ThermoFisher Scientific) diluted 1:2000, and SuperSignal™ West Pico Chemiluminescent Substrate kit (34077, ThermoFisher Scientific). Total protein concentration was assessed by the use of Protein Quantification Assay (740967, Macherey-Nagel GmbH & Co.) and CLARIOstar BMG Labtech. Equal loading of proteins (20 μg) was verified by antibody recognizing β-actin (A2066, ThermoFisher Scientific 1:500). Images were obtained using the ImageQuant™ LAS-4000 imagine system (GE Healthcare Life Science).

### SAGE sequencing

Postsurgical tissue specimens were immediately submerged in liquid nitrogen and stored at −80 °C until further processing. In total, 23 tumor specimens with paired normal tissue samples were used in SAGE library preparations. RNA for SAGE-sequencing was extracted using RNeasy Fibrous Tissue Mini Kit (74704, Qiagen), following isolation by Trizol reagent (15596026, ThermoFisher Scientific) according to the manufacturer’s protocol. Only RNA samples of high quality (RNA Integrity number (RIN) ≥7.0) were used in library preparations. SAGE libraries were prepared following the SOLiD SAGE Kit with Barcoding Adaptor Module Guide (4452811, ThermoFisher Scientific), using restriction endonucleases Nla III and EcoP15I. Different SAGE libraries were barcoded and pooled together for emulsion PCR to a total concentration of 0.5 pM. Sequencing was performed on two full SOLiD™ 6-lane FlowChips using SOLiD 5500xl sequencer at the Nord University (Bodø, Norway). Sequencing generated about 28 billion nucleotides (16–99 million reads; Supplementary Table S2). SAGE tags corresponding to the size of 26- and 27-bp were extracted from the raw reads and uniquely mapped in color space to human genome reference GRCh38 using bowtie^50^, allowing for maximum three mismatches. Counting of reads was performed using HTSeq^51^ without strand specificity (–stranded = no) and with union overlap resolution mode. Differential expression analyses were performed by the DESeq2 package^52^ with a FDR cutoff of 0.05. Additional figures were created using R.

### Statistical analyses

GraphPad Prism software was used to perform statistical comparison analyses. Results are expressed as mean ± SEM. Statistical significance was set at *p* < 0.05. Statistical significance was assessed using a Paired t test, one-way or two-way analysis of variance (ANOVA) followed by the Bonferroni’s Multiple Comparison correction posttest (Prism version 5.0; GraphPad Software) as indicated in the figures. Statistical significance is indicated in the figures as follows: ***p < 0.001; **p < 0.005; *p < 0.05; and ns, non-significant (*p* > 0.05).

## Electronic supplementary material

Supplementary information

Dataset1

Dataset2
