# Supplementary material for: ICAM1 expression is induced by proinflammatory cytokines and associated with TLS formation in aggressive breast cancer subtypes
Source: Sci Rep. 2018 Aug 6;8:11720. doi: 10.1038/s41598-018-29604-2 (PMC6079003; doi:10.1038/s41598-018-29604-2)
Supplement: Supplementary file 1 — Supplementary information [file 41598_2018_29604_MOESM1_ESM.pdf]

TITTLE PAGE

ICAM1 expression is induced by proinflammatory cytokines and associated with TLS formation in aggressive breast cancer subtypes.

Stine L. Figenschau[1], Erik Knutsen[1], Ilona Urbarova[2], Christopher Fenton[3], Bryan Elston[4], Maria Perander[1], Elin S. Mortensen[1,5], Kristin A. Fenton[1]

[1] RNA and Molecular Pathology Research group, Department of Medical Biology, Faculty of Health Science, UiT – The Arctic University of Norway, N-9037 Tromsø, Norway

[2] Tromsø University Proteomics Platform, Department of Medical Biology, Faculty of Health Science, UiT – The Arctic University of Norway, N-9037 Tromsø, Norway

[3] The Microarray Platform, Faculty of Health Science, UiT – The Arctic University of Norway, N-9037 Tromsø, Norway

[4] Histopath Diagnostic Specialists, Suite 201, 4 Drake Avenue, Building B Pinnacle Office Park, Macquarie Park NSW 2113, Australia

[5] Department of Clinical Pathology, University Hospital of North Norway, N-9038 Tromsø, Norway

Correspondence to:

Kristin A. Fenton, RNA and Molecular Pathology Research Group, Department of Medical Biology, Faculty of Health Sciences, UiT - The Arctic University of Norway, MH-building, 9037 Tromsø, Norway.

Phone: +4777664834.

E-mail: kristin.fenton@uit.no

Table S1. R script for differential expression analysis in DESeq2

Tumor vs. Normal:

```
#load counts
counts <- read.table("counts.tab", header=T, row.names=1, sep="\t")

#clean pheno file
pheno <- rep("N", ncol(counts))
pheno[grep("T", colnames(counts))] <- "T"

#sync counts with pheno
#remove empty reads
counts <- counts[which(rowSums(counts) > 0),]

pheno <- cbind("ID"=colnames(counts), "pheno"=pheno)
rownames(pheno) <- pheno[,1]

#DEseq2
library(DESeq2)

dds <- DESeqDataSetFromMatrix(countData = counts, colData=pheno, design = ~
pheno)
res <- results(dds, lfcthreshold=2) #logFoldChange greater or around 2
res <- res[which(res$baseMean > 10),] #remove low count transcripts
res <- res[which(res$padj < 0.05),] #only those that have a padj less than
0.05
res <- res[order(res$padj),] #order by padjust

library(gProfileR)

#annotate
xx <- gconvert(rownames(res), organism = "hsapiens", target = "ENSG",
region_query = F,numeric_ns = "",
mthreshold = Inf, filter_na = T, df = T)
dups <- which(duplicated(xx[,2]))
xx <- xx[-dups,]
rownames(xx) <- tolower(xx[,2])
test <- as.data.frame(res)
dd <- cbind(test, xx[rownames(test),])

write.table(dd, "kristin_tumour_normal.tab", sep="\t")
png("kristin_up_down.png")
plotCounts(dds, "uc003thr.4", intgroup="pheno")
dev.off()
```

Table S2. R script for differential expression analysis in DESeq2

TLS pos vs. negative:

```
#load counts
counts <- read.table("counts.tab", header=T, row.names=1, sep="\t")
#clean pheno file
pheno           <- read.table("New_groups.txt", header=T, sep="\t")
pheno           <- pheno[!is.na(pheno[, ncol(pheno)]),]
rownames(pheno) <- paste(paste("x", pheno$ID, sep=""), "T", sep="")
rownames(pheno) <- gsub("xT", "x", rownames(pheno))
#sync counts with pheno
counts <- counts[, rownames(pheno)]
#remove empty reads
counts <- counts[which(rowSums(counts) > 0),]
#DEseq2
library(DESeq2)
pheno$Nye.Grupper <- as.factor(pheno$Nye.Grupper)
dds                             <- DESeqDataSetFromMatrix(countData = counts,
colData=pheno, design = ~ Nye.Grupper)
#normalized principle component
library(matrixStats)
library(genefilter)
ntop   <- 500
rld    <- rlog(dds, blind=FALSE)
rv     <- rowVars(assay(rld))
select <- order(rv, decreasing = TRUE)[seq_len(min(ntop,length(rv)))]
pca    <- prcomp(t(assay(rld)[select, ]))
colors <- as.character(pheno[rownames(pca$x), "Nye.Grupper"]) colors[colors ==
"1"] <- "blue"
colors[colors == "2"] <- "red"
png("normalized_pca_inflammed_vs_non_tumour.png", width=800, height=800)
plot(pca$x[,1], pca$x[,2], col=colors, pch=20) labels <-
paste(rownames(pca$x), pheno[rownames(pca$x), "Nye.Grupper"], sep="_")
text(pca$x[,1], pca$x[,2], labels, pos=2)
dev.off()
#dds analysis
dds   <- DESeq(dds)
res   <- results(dds)
sig   <- res[which(res$pvalue < 0.05),] #too many
sig   <- sig[which(sig$padj < 0.1),]    #too few with padj 0.05
sig   <- sig[which(sig$baseMean > 10),] #too many with very low means
#annotate
library(gProfileR)
xx    <- gconvert(rownames(sig), organism = "hsapiens", target =
"ENTREZGENE_ACC", region_query = F,numeric_ns = "", mthreshold = Inf,
filter_na = T, df = T)
annot <- cbind(sig[as.character(tolower(xx[,2])),], xx)
path  <- gprofiler(unique(as.character(xx$name)), organism = "hsapiens")
#results
write.table(annot, "DEseq_inflammed_non_inflammed_tumour.txt", sep="\t")
write.table(path, "DESeq_inflammed_pathways.txt", sep="\t")
```

Table S3. The amount of reads gained from SAGE sequencing.

Sample name	No. of raw reads [1]	No. of mapped SAGE tags [2]	% of mapped SAGE tags [3]
SAGE_1T	24,258,091	15,635,747	64.5
SAGE_3T	31,994,239	22,032,673	68.9
SAGE_4T	27,083,634	17,166,279	63.4
SAGE_6T	25,124,284	17,469,710	69.5
SAGE_7T	28,390,731	18,592,583	65.5
SAGE_9T	22,038,727	15,183,800	68.9
SAGE_10T	23,928,559	15,617,125	65.3
SAGE_11T	19,578,639	13,898,273	71.0
SAGE_12T	17,530,938	10,975,834	62.6
SAGE_13T	14,968,593	10,050,627	67.1
SAGE_15T	16,579,277	10,450,145	63.0
SAGE_16T	23,487,429	15,651,523	66.6
SAGE_17T	18,818,245	13,272,280	70.5
SAGE_18T	18,570,125	13,024,410	70.1
SAGE_20T	23,858,957	15,148,028	63.5
SAGE_21T	28,704,826	20,521,979	71.5
SAGE_22T	24,963,695	17,859,750	71.5
SAGE_24T	22,028,079	15,559,328	70.6
SAGE_25T	20,377,702	15,245,459	74.8
SAGE_26T	40,919,575	31,972,672	78.1
SAGE_27T	25,596,230	14,629,812	57.2
SAGE_30T	18,867,822	13,284,918	70.4
SAGE_31T	15,744,015	8,997,525	57.2
SAGE_1N	45,402,414	26,550,066	58.5
SAGE_3N	36,374,516	25,581,619	70.3
SAGE_4N	39,144,024	27,277,375	69.7
SAGE_6N	26,433,945	16,552,837	62.6
SAGE_7N	32,676,707	23,166,747	70.9
SAGE_9N	36,049,982	25,041,739	69.5
SAGE_10N	35,718,425	26,435,374	74.0
SAGE_11N	29,510,280	18,878,549	64.0
SAGE_12N	33,231,009	24,714,464	74.4
SAGE_13N	27,230,170	19,016,580	69.8
SAGE_14N	27,595,512	18,745,805	67.9
SAGE_15N	18,915,521	12,325,515	65.2
SAGE_16N	16,193,475	11,207,480	69.2
SAGE_17N	27,621,901	18,502,102	67.0
SAGE_18N	20,753,933	14,083,017	67.9
SAGE_20N	20,130,803	12,262,315	60.9
SAGE_21N	22,473,505	12,455,426	55.4
SAGE_22N	19,623,149	13,774,768	70.2
SAGE_23N	19,750,852	13,249,316	67.1
SAGE_24N	17,813,930	11,789,423	66.2
SAGE_25N	26,411,798	17,838,380	67.5
SAGE_26N	99,480,765	71,968,531	72.3
SAGE_27N	23,354,973	17,036,342	73.0
SAGE_30N	29,471,098	16,642,362	56.5
SAGE_31N	25,819,634	18,290,879	70.8

[1] Number of raw reads gained by SOLiD SAGE sequencing.

[2] Number of 26bp tags mapping to human genome reference GRCh38 in color space using bowtie.

[3] Percentage of SAGE tags mapped from the amount of raw reads gained by SAGE sequencing.

Figure S1

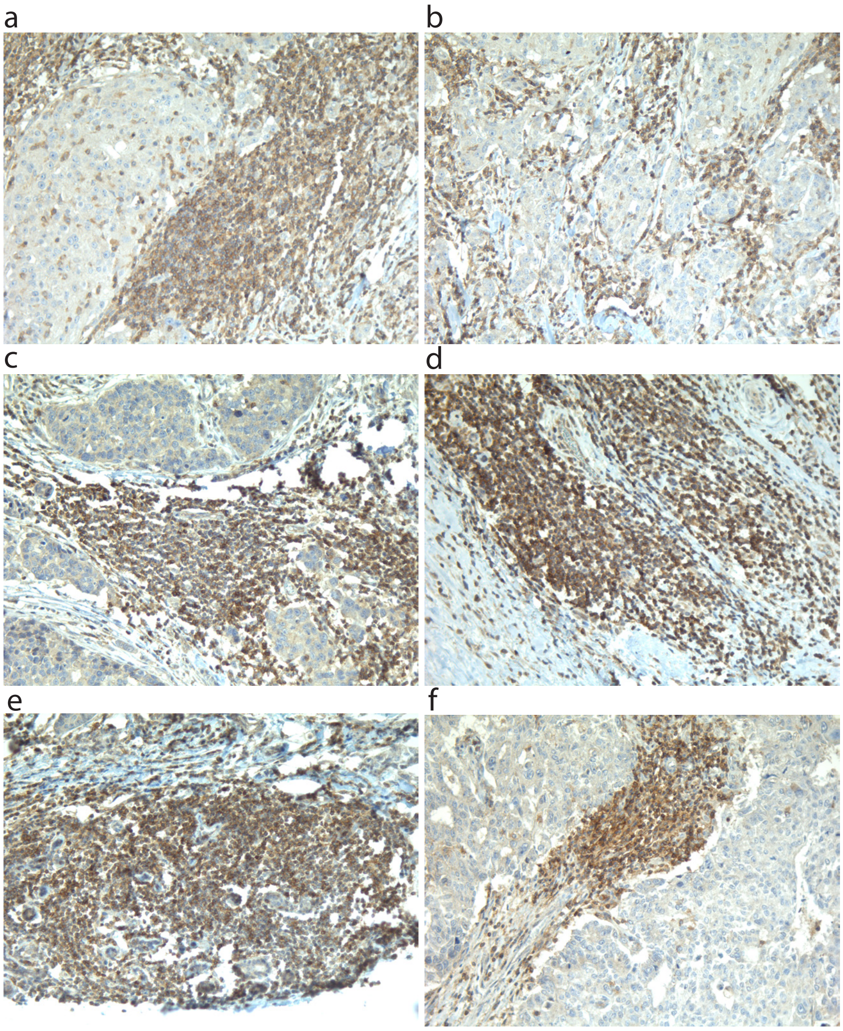

Figure S1: ITGAL expression in breast cancer patients.

Tumor sections from TNBC patients showing ITGAL immunoreactivity in **(a)** immune cell aggregates in close proximity to the tumor cells, and **(b)** in tumor-associated stroma. Prominent ITGAL expression was detected in tumor-associated TLS both **(c, d)** in the central tumor and **(e, f)** the periphery of the tumor. Original magnification, X200.

Figure S2

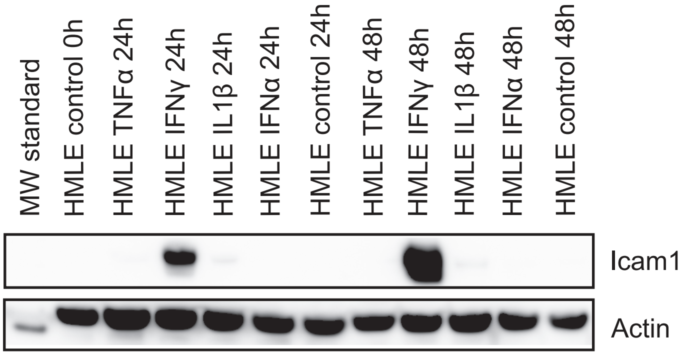

a **b**

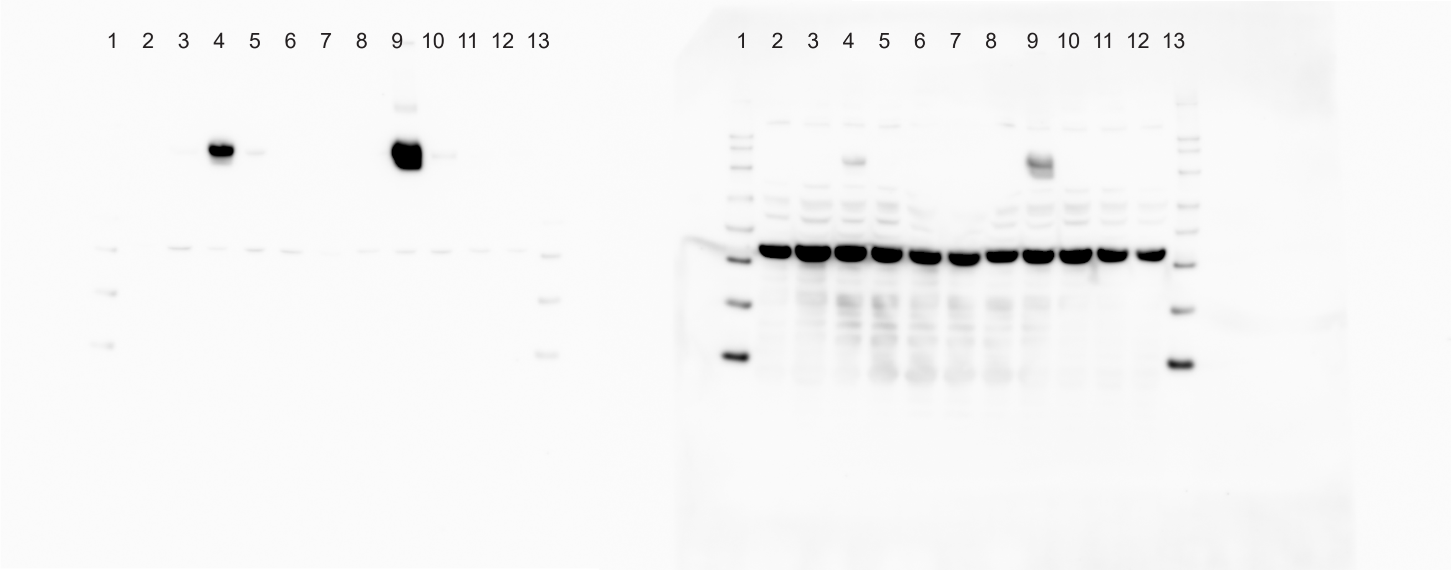

Figure S2. Full-length membranes of ICAM1 expression in HMLE cells after stimulation with proinflammatory cytokines. (**a**) Lane 1 and 13: Magic marker MW standard. Lane 2: control after 0h. Lane 3: TNFα 24h. Lane 4: IFNγ 24 h. Lane 5: IL1β 24h. Lane 6: IFNα 24h. Lane 7: Control 24h. Lane 8: TNFα 48h Lane 9: IFNγ 48 h. Lane 10: IL1β 48h. Lane 11: IFNα 48h. Lane 12: control 48h. (**b**) Actin beta of the membrane in **a**.)

Figure S3

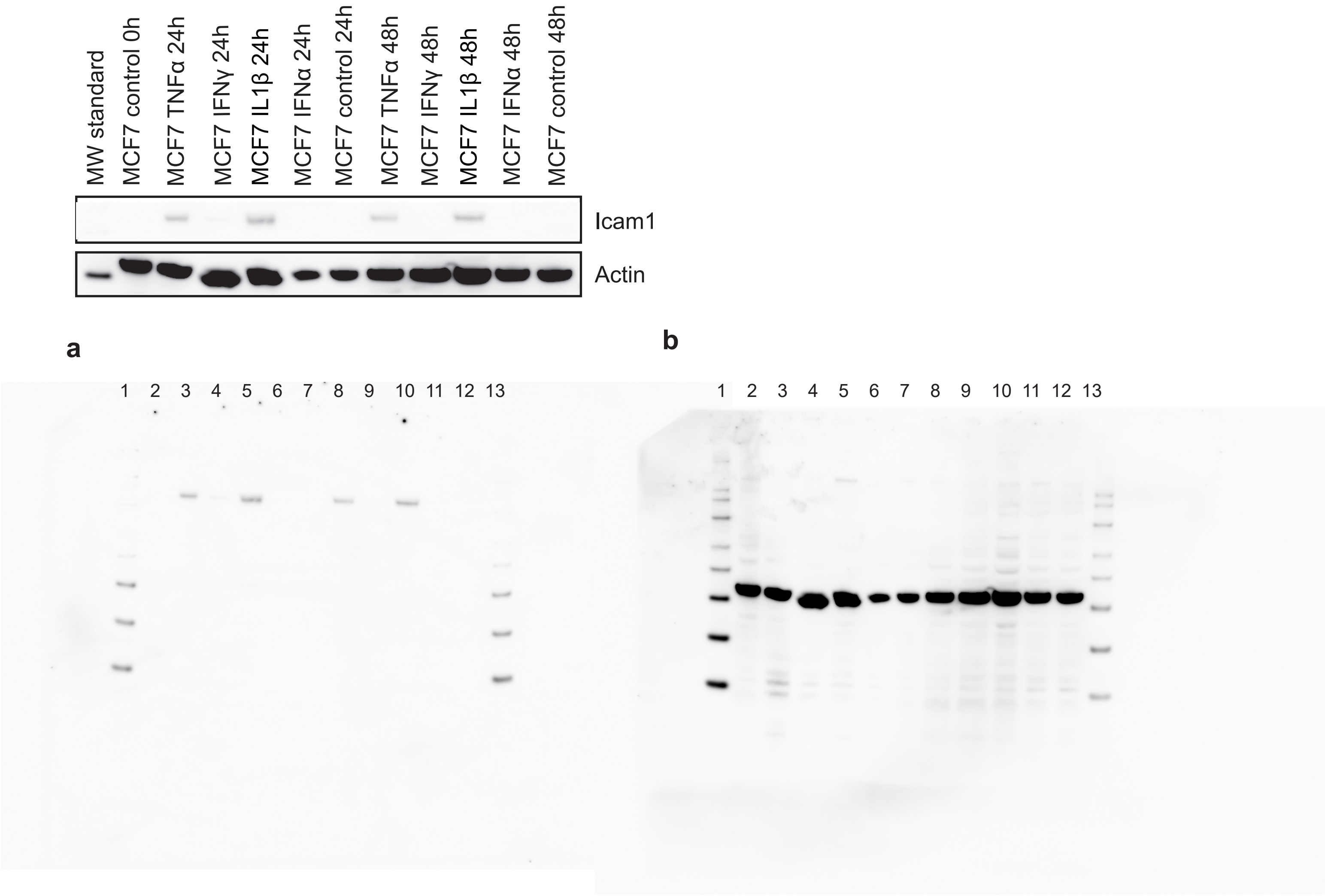

Figure S3. Full-length gels of ICAM1 expression in MCF7 cells after stimulation with proinflammatory cytokines. (**a**) Lane 1 and 13: Magic marker MW standard. Lane 2: nonstimulated MCF7 cells after 0h. Lane 3-6: MCF7 cells stimulated with TNFα (lane 3), IFNγ (lane 4), IL1β (lane 5) and IFNα (lane 6) for 24h. Lane 7: nonstimulated MCF7 cells at 24h. Lane 8-11: MCF7 cells stimulated with TNFα (lane 8), IFNγ (lane 9), IL1β (lane 10) and IFNα (lane 11) for 48h. Lane 12: nonstimlated MCF7 cells at 24h (**b**) Actin beta detection of the membrane in **a**.

Figure S4

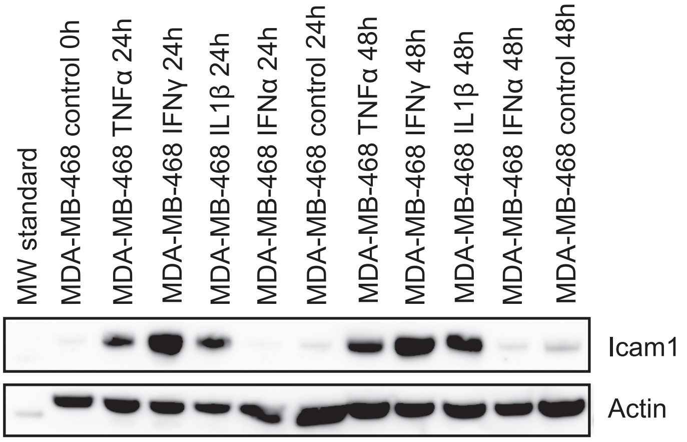

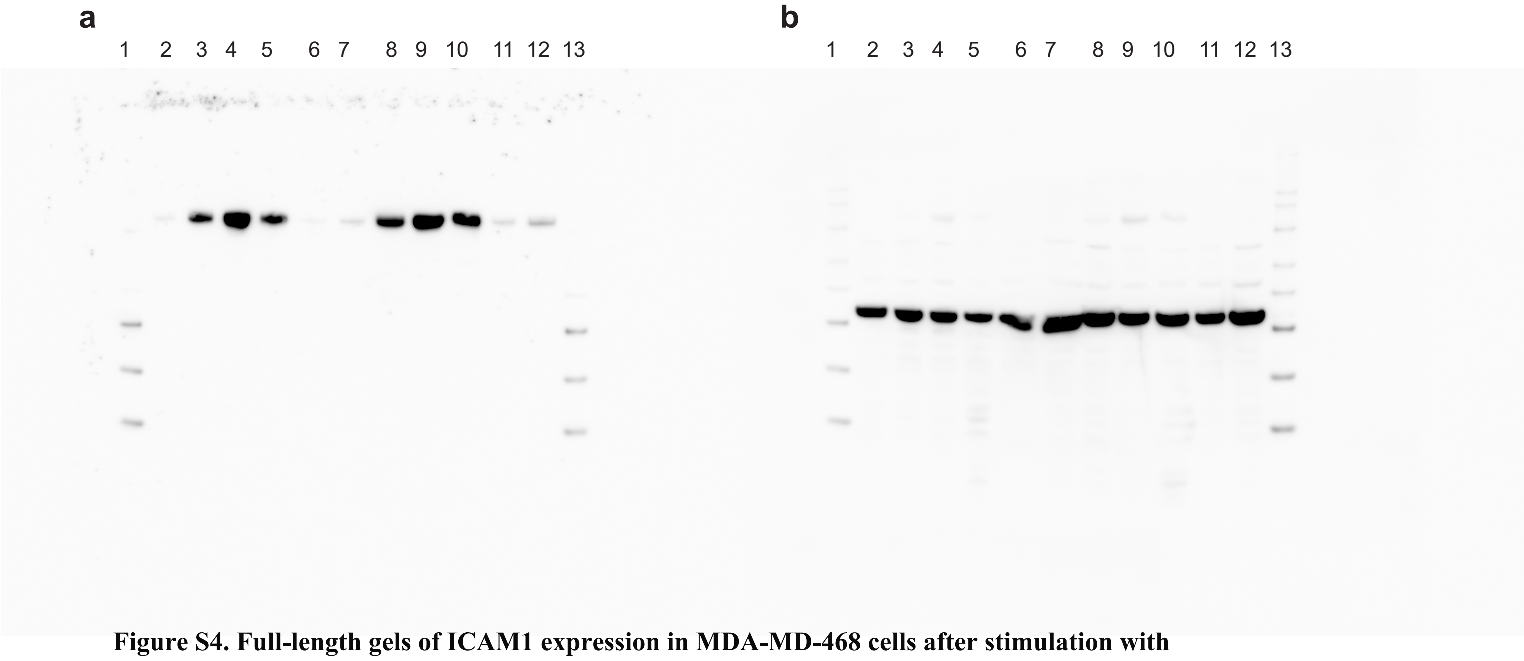

Figure S4. Full-length gels of ICAM1 expression in MDA-MD-468 cells after stimulation with proinflammatory cytokines. (**a**) Lane 1 and 13: Magic marker MW standard. Lane 2: nonstimulated MDA-MD-468 cells after 0h. Lane 3-6: MDA-MD-468 cells stimulated with TNFα (lane 3), IFNγ (lane 4), IL1β (lane 5) and IFNα (lane 6) for 24h. Lane 7: nonstimulated MDA-MD-468 cells at 24h. Lane 8-11: MDA-MD-468 cells stimulated with TNFα (lane 8), IFNγ (lane 9), IL1β (lane 10) and IFNα (lane 11) for 48h. Lane 12: nonstimulated MCF7 cells at 48h (**b**) Actin beta detection of the membrane in **a**.

Figure S5

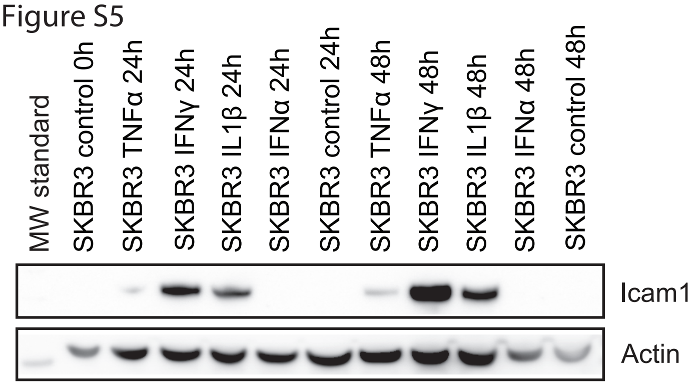

a

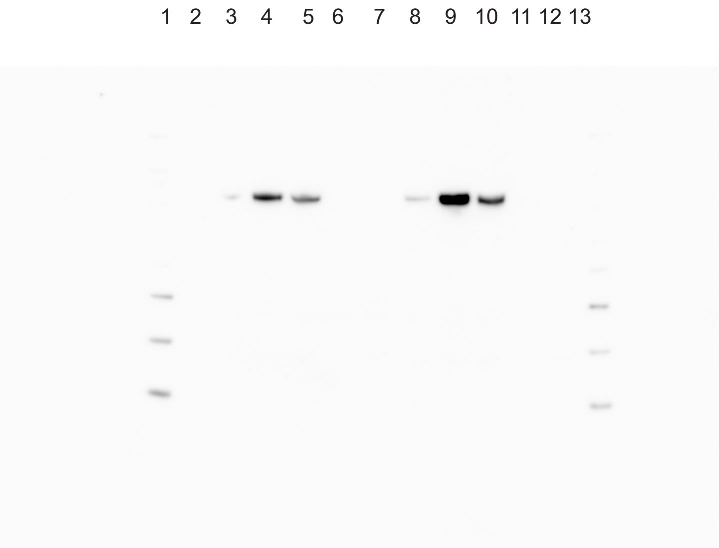

b

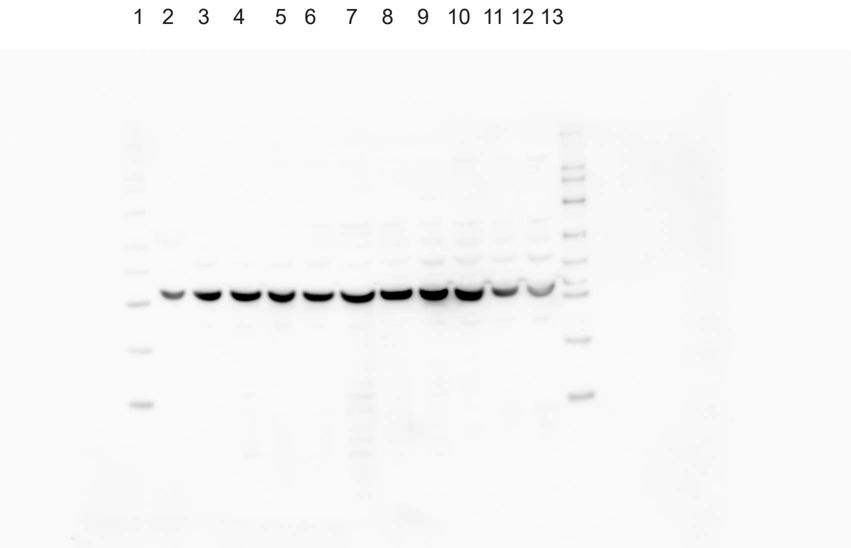

Figure S5. Full-length gels of ICAM1 expression in SKBR3 cells after stimulation with proinflammatory cytokines. (**a**) Lane 1 and 13: Magic marker MW standard. Lane 2: nonstimulated SKBR3 cells after 0h. Lane 3-6: SKBR3 cells stimulated with TNFα (lane 3), IFNγ (lane 4), IL1β (lane 5) and IFNα (lane 6) for 24h. Lane 7: nonstimulated SKBR3 cells at 24h. Lane 8-11: SKBR3 cells stimulated with TNFα (lane 8), IFNγ (lane 9), IL1β (lane 10) and IFNα (lane 11) for 48h. Lane 12: nonstimulated SKBR3 cells at 48h (**b**) Actin beta detection of the membrane in **a**.
